# Dynamics of Language Learning Motivation and Emotions: A Parallel-Process Growth Mixture Modeling Approach

**DOI:** 10.3389/fpsyg.2022.899400

**Published:** 2022-06-21

**Authors:** Hanjing Yu, Hongying Peng, Wander M. Lowie

**Affiliations:** ^1^School of Foreign Language, Dalian University of Technology, Dalian, China; ^2^Center for Linguistics and Applied Linguistics, Guangdong University of Foreign Studies, Guangzhou, China; ^3^Department of Applied Linguistics, University of Groningen, Groningen, Netherlands

**Keywords:** language learning motivation, emotions, salient patterns, adaptive interactions, a parallel-process GMM approach

## Abstract

The present study adopted a novel parallel-process growth mixture modeling (GMM) technique to research the adaptive interaction between foreign language learners’ learning motivation and emotions, with a view to advancing our understanding of how language learning motivation and emotions (enjoyment and anxiety) adaptively interact with each other over time. The present study, situated in the Chinese English as a foreign language (EFL) learning context, collected learning motivation and emotion data from 176 Chinese EFL learners over a period of two semesters (12 months). The GMM technique adopted in the study identified three developmental profiles of motivation and two of emotions, respectively. The study further distilled salient patterns of motivation–emotion interaction over time, patterns significant for designing and implementing pedagogical interventions for motivation enhancement. The parallel-process GMM technique was also proven to be a useful approach to parsing learner variety and learning heterogeneity, efficiently summarizing the complex, dynamic processes of motivation and emotion development.

## Introduction

An increasing number of researchers have embraced a dynamic and fluid view of motivation for second and foreign language (L2) learning (Dörnyei et al., 2015), and adopted the Complex Dynamic Systems Theory (CDST) as a research paradigm. Existing CDST-based motivation research has shed valuable light on the dynamic and idiographic nature of L2 learning motivation; that is, L2 learners’ learning motivation is likely to change over time and the process of motivation development tends to be specific to the individual (e.g., Chan et al., 2015; Waninge, 2015; Teimouri, 2017; Papi and Hiver, 2020). Albeit with this individual heterogeneity, researchers (e.g., Dörnyei, 2010; Zheng et al., 2020; Peng et al., 2022) have recently begun to realize that there might still exist typical developmental patterns of motivation, which are often shared by different individuals, observable at a relatively global level (e.g., at the group/subgroup level), and detectable with advanced modeling techniques.

Identifying such typical motivational patterns can advance our understanding of the nature and development of language learners’ learning motivation, illustrating some “nomothetic knowledge about idiographic processes” of motivation development (Molenaar, 2015, p. 40). Specifically, the patterned outcomes regarding motivation development have the potential to increase the generalizability of CDST-based motivation research findings. Meanwhile, different learner types, each with a distinct motivational pattern, might also be revealed. With the identification of salient motivational patterns and typical learner types, it would be possible to provide pedagogical suggestions helpful for educators to design and implement instructional interventions tailored to different learners, strategically and differently enhancing their motivation development. This is particularly important for English as a foreign language (EFL) learning contexts (e.g., Chinese EFL learning context) where each language teaching class is made up of a large number of students who need to be motivated and scaffolded differently. Uncovering different learner types with distinct patterns of motivation development may have the potential to help educators boost and modify language learners’ learning motivation differently and properly.

Situated in the Chinese EFL learning context, the present study proposes a novel parallel-process growth mixture modeling (GMM) approach to identifying the salient trajectories (showing distinct development patterns) of 176 college-level Chinese EFL learners’ motivation changes over a period of two semesters. In doing so, it is hoped to provide a macro-level map of Chinese EFL learners’ motivation development over time. More specifically, the present study contributes to L2 motivation literature in three important ways: (1) theoretically, it generates new insights into the nature and mechanism of motivation by way of identifying salient developmental patterns that transcend individual heterogeneity; (2) methodologically, with the application of the parallel-process GMM technique, it adds to the analytic toolkit for our investigation into the signature dynamics of L2 learners’ learning motivation; and (3) pedagogically, it provides practical suggestions for the design and implementation of instruction strategies tailored for different learners with distinct developmental patterns of motivation.

Another goal of the study is to further explore the adaptive interaction between language learners’ learning motivation and emotion over time, two factors both essential for L2 learning (Dewaele, 2012; Ryan, 2019) and recently found to be closely related to each other (Teimouri, 2017; Saito et al., 2018). Specifically, the present study explores the ways in which different emotions (enjoyment and anxiety) would exert different influences on Chinese EFL learners’ learning motivation over time, distilling patterns of the temporal connection and adaptive interaction between the two, and hence advancing our understanding of the mechanisms that potentially shape the complex and dynamic process of language learners’ motivation development.

## Literature Review

### Dynamics Processes of Motivation and Emotion

#### Motivation

With a growing awareness of the dynamic, interconnected feature of language learning motivation, more and more studies have adopted the CDST perspective (with its accompanying analytic techniques) to researching learning motivation, and yielded insightful findings regarding the complexity and dynamics of motivational processes, as well as developmental patterns of motivation (e.g., Chan et al., 2015; Dörnyei and Ryan, 2015; Piniel and Csizér, 2015; Al-Hoorie, 2019; Papi and Hiver, 2020; Zheng et al., 2020). For example, in the first volume dedicated to the dynamics of motivation, *Motivational Dynamics in Language Learning*, Piniel and Csizér (2015) investigated Hungarian freshman students’ motivational changes over a period of one semester. Guided by the L2 Motivation Self System (L2MSS) framework (Dörnyei, 2009), and adopting a latent growth curve modeling technique (LGCM) and a cluster analysis technique, the study found small but unique fluctuations in Hungarian students’ learning motivation over time. Specifically, two dimensions (i.e., the ought-to L2 self and the L2 learning experience) of the L2MSS framework were found most likely to change, while the ideal L2 self-dimension and students’ motivated learning behaviors remained relatively stable over time.

Research has also been conducted to investigate patterned outcomes beyond the individual idiosyncrasy. For instance, Zheng et al. (2020) investigated the evolution and adaptation of Chinese learners’ motivation to learn a third language (L3 Spanish learning in addition to L2 English) over 1.5 years using Q methodology. Patterned outcomes—two different profiles—regarding learners’ motivational changes were found. One profile has a dominating translingual and transcultural orientation that develops into more constitutive ideal multilingual selves, while the other has a dominating instrumental orientation that generates decreasing motivational forces.

More recently, using a GMM technique, Guay et al. (2021) identified the salient patterns of motivation development that emerged from Canadian secondary school learners’ motivational dynamics over time. In line with the self-determined theory (SDT), Guay et al. (2021) included different motivational variables in the study, based on which they identified five different motivation profiles (i.e., low, high-stable, increasing, moderate, and high). Findings of this research provided new insights into motivational dynamics, enriching our understanding of the essential similarities and differences across individual dynamics of motivation development (i.e., identifying *inter-individual differences in intra-individual changes* over time; see also Peng et al., 2022).

Although those studies have provided important insights into the patterned outcomes regarding learners’ motivational dynamics, they are still limited in literature. Moreover, existing exploration of motivational patterns paid limited attention to language learners’ motivational dynamics, particularly less in the EFL learning context, a context in urgent need of identifying the salient profiles shared by different learners for tailored pedagogical support, as aforementioned. As such, the present study continues the exploration of salient patterns of learners’ motivational dynamics, under the L2MSS framework—a framework more commonly adopted in L2 research and closely in line with the CDST perspective adopted in the present study (Dörnyei, 2009). Specifically, the study examines whether and to what extent typical patterns would emerge from 176 Chinese EFL learners’ processes of motivation development.

#### Emotion

Another goal of the present study is to explore the ways in which emotions (enjoyment and anxiety) would affect EFL learners’ learning motivation over time. As indicated in previous studies (Teimouri, 2017; Saito et al., 2018), language learners’ learning motivation is often closely related to their emotions, and, from a CDST perspective, learners’ motivation and emotions (e.g., anxiety and enjoyment) function in a co-adaptive manner. For instance, anxiety, the most extensively studied emotion in the field of second language acquisition (SLA; Dewaele, 2007; MacIntyre, 2017; Teimouri, 2017; Shao et al., 2019, 2020), has been fruitfully conceptualized and validated as a negative factor detrimental to learners’ learning motivation and to their ultimate language achievement (Horwitz, 2010; Papi, 2010; Dewaele and MacIntyre, 2016; Teimouri, 2017).

More recently, research attention has been shifted toward positive emotions (e.g., enjoyment) and their roles as a driving force for language learning and development (e.g., MacIntyre and Gregersen, 2012; Dewaele and MacIntyre, 2014). This shift in research attention is ascribable to the observation that “positive emotion has a different function from negative emotion; they are not opposite ends of the same spectrum” (MacIntyre and Gregersen, 2012, p. 193). To be specific, learners who enjoy their language learning are expected to better acquire and develop a foreign/second language (Dewaele and Alfawzan, 2018). As a result, researchers (e.g., Dewaele and MacIntyre, 2014, 2016; Dewaele et al., 2016; Dewaele and Dewaele, 2017) are increasingly taking both negative and positive emotions into consideration in order to analyze language learners’ emotion in a relatively comprehensive manner. For example, Dewaele and MacIntyre (2014) investigated foreign language learners’ anxiety and enjoyment (representative of a negative and a positive emotion, respectively) in the classroom, and found that levels of enjoyment were significantly higher than those of anxiety.

### Adaptive Interaction Between Motivation and Emotion

The close connection and adaptive interaction between learning motivation and emotion can find theoretical support in the CDST framework. That is, according to the CDST perspective, different variables (e.g., learning motivation and emotion) would interact intricately and adaptively with each other, jointly shaping learners’ language learning and developmental processes (Larsen-Freeman and Cameron, 2008). Inherent in the intricate interaction are self-organization and co-adaptation, two central features of the CDST perspective. Self-organization refers to a process of a system adapting its internal structure or function in response to external circumstances (Dekker et al., 2011), while co-adaptation indicates a process in which components (or variables) of a system “constantly reorganize their internal working parts to adapt themselves to the problems posed by their surroundings [or by other components]” (Hiver and Al-Hoorie, 2020, p. 26). Through self-organizing and co-adapting, learners’ learning motivation and emotion maximize the functioning of the integrated motivation system, leading to coordinated behavior between the two variables (Jirsa and Kelso, 2004). This motivation–emotion adaptation not only describes a fruitful repertoire for language learners’ learning behavior (Lowie and Verspoor, 2019), but also further “illustrates how simplicity arises from the adaptive behavior of interrelated and interacting components” (Papi and Hiver, 2020, p. 213).

Empirical evidence has been yielded with regard to the adaptive interaction between motivation and emotion. For instance, Papi and Teimouri (2014) identified six L2 learner motivational types characterized by distinct combination and interaction of motivational, emotional, and linguistic variables, contributing to L2 motivation research theoretically and pedagogically. Moreover, a positive relationship between motivation and anxiety was also spotted in one specific learner type. In another study, Teimouri (2017) investigated how learners’ emotions related to their L2 selves under the L2MSS framework, and a positive path from enjoyment to motivation was detected.

So far, studies that investigated the interplay between motivation and emotion have mainly been cross-sectional in nature. Longitudinal studies have been rare. To date, only Pan and Zhang (2021) adopted a longitudinal design to investigate how learners’ emotions (enjoyment and anxiety) developed over time, and how learners’ learning motivation interacted with their emotion and how the motivation–emotion relation changed over time. The results showed that, when compared to anxiety, enjoyment was less stable over time. Additionally, some motivational factors (e.g., ideal L2 self and ought-to L2 self) were found in close relation to the changes in enjoyment and anxiety. Although those studies have broadened our understanding of the interrelationship between learning motivation and emotions, longitudinal research, a more fruitful way of providing insights into the adaptive interaction between different variables over time and into the influence these variables exert on each other, is rare and much needed (DeKeyser, 2012; Piniel and Csizér, 2015). Also needed are innovative modeling-based techniques with strong validity to explore the cooperative and adaptive interaction between motivation and emotion over time.

### Parallel-Process GMM Technique

The present study adopts a GMM technique, a modeling-based technique with strong validity (Warschauer et al., 2019), which has also been recently applied in general learning motivation research (e.g., Guay et al., 2021; Lee and Ju, 2021). GMM as an extension of the latent growth curve model (LGCM; Bollen and Curran, 2006) specifies two parameters (i.e., intercept and slope) that can capture the general trend and temporal changes of individual development over time. Unlike LGCM assuming only one homogeneous population present in the chosen sample (Duncan et al., 2006), GMM provides an appropriate way of uncovering “inter-individual differences in intra-individual change [while] taking into account unobserved heterogeneity within a larger population” (Jung and Wickrama, 2008, p. 303). Specifically, the GMM technique operates in a bottom-up manner, examining individual-level motivational processes over time first, and then exploring the existence of similarly structured individuals (i.e., individuals with shared developmental patterns of motivation over time). This bottom-up procedure is consistent with the CDST approach in that attention is first paid to the individual-level developmental dynamics and then shifted to the salient patterns that hold across individuals, patterns that emerge from the data and transcend the individual heterogeneity (see Bulté and Housen, 2020; Peng et al., 2022).

Applied to the motivation and emotion research, GMM is capable of identifying and depicting the typical patterns of motivational and emotional changes over time (patterns shared by different individuals). Further, by adopting a parallel-process GMM technique, it is possible to examine whether the patterned outcomes regarding developmental trajectories of motivation are associated with those of emotion, delving deeper into the temporal cooperation, and adaptive interaction between motivation and emotion over time (Muthén, 2001).

To date, only one study (Lee and Ju, 2021) adopted the parallel-process GMM technique to investigate general learning motivation. Lee and Ju (2021) explored the motivation trajectories of South Korean learners, based on three components of extrinsic motivation and competence beliefs. The study identified three different patterns of developmental trajectories for external regulation, and four distinct classes for introjected and identified regulation, respectively. They further explored how competence beliefs affected motivation development over time, providing a holistic and more nuanced picture of the development of multidimensional motivation.

The present study, guided by the CDST perspective, similarly adopts the innovative parallel-process GMM technique to research foreign language learners’ learning motivation, with a view to detecting the salient patterns of Chinese EFL learners’ motivation development, and to further exploring its dynamic and adaptive interplay with learners’ emotions over time. The study provides added value in that, first, it is situated in the Chinese EFL learning context. Previous research on motivation–emotion interaction was mainly conducted among English as a second language (ESL) learners. It would be necessary to also investigate in what ways EFL learners’ motivation coordinate and interact with their emotions over time, an issue essential for making a more generalized interpretation of the relationships between motivation and emotion for larger population. Second, it explores the salient patterns of the motivational dynamics under the L2MSS framework—a framework closely in line with the CDST perspective adopted in the present study.

## The Present Study

Through a novel parallel-process GMM method, the present study is intended to capture the salient trajectories of 176 Chinese EFL learners’ motivation and emotion development, and to explore the temporal and dynamic interplay between motivation and two distinct emotions (i.e., anxiety and enjoyment), respectively, over a period of two semesters. Specific research questions are:

*RQ1*. Can we identify salient trajectories of motivation and emotions (anxiety and enjoyment), respectively, among 176 Chinese EFL learners?
*RQ2*. How is Chinese EFL learners’ motivation related to their emotions (anxiety and enjoyment) over time?

### Participants

The participants of the present study were 176 (52 females, 124 males) non-English major freshmen from five parallel classes at a University in Northern China. Based on the instructions proposed by the Ministry of Education of the People’s Republic of China, Chinese students would take the *Gaokao* (National College Entrance Examination, NCEE) by the end of senior school years, in which English is one of the three main subjects (each subject accounting for 150 scores). The NCEE scores are considered a valid college admission criterion. Participants of the present study had an average NCEE score of 129 (*Range* = 44, *SD* = 8.62). After joining different faculties of the university, students would take English placement tests through Speexx and be automatically assigned to different levels of English courses based on their testing scores. Speexx is an online language learning and testing platform that screens language levels of learners based on Common European Framework of Reference for Language (CEFR). The participants in this study were awarded the beginning level of proficiency (CEFR B1.1). At the start of this research project, the participants enrolled in a reading course instructed by the first author once a week. The course lasted for two semesters. Throughout the reading course, the first author adopted the same teaching strategies with the same reading textbook in the five parallel classes. Specifically, students learnt one text with different themes every 2 weeks, and discussed the topics that include main idea, supporting, details, the purpose of the author, fact and opinion, organizational patterns, relationships, vocabulary in context, inference and conclusions, reasoning, and argument.

### Measures

The questionnaire included 35 items that assess learners’ motivation and emotions (positive and negative). The items included were mainly adapted from previous research instruments (e.g., Dewaele and MacIntyre, 2014; Papi and Teimouri, 2014). The measure of the items was scored on a six-point Likert scale, with 1 indicating *strongly disagree* or *not at all* and 6 referring to *strongly agree* or *very much*. The questionnaire had been piloted and successfully applied in Peng et al. (2020). It consisted of two parts:

Part I collected participants’ background information such as gender, educational level, and language proficiency (NCEE score).
Part II included items that measured learners’ motivational and emotional states, and the motivation in the present study was operationalized in line with L2MSS of Dörnyei (2009). The variables (Cronbach Alpha reliability indexes calculated and reported) indicative of learner motivation and emotion included ideal L2 self, ought-to L2 self, L2 learning experience, enjoyment, and anxiety:
*Ideal L2 self* (five items, *α*_time1_ = 0.889; *α*_time2_ = 0.911; *α*_time3_ = 0.936; *α*_time4_ = 0.942; *α*_time5_ = 0.948; and *α*_time6_ = 0.935), measures learners’ own aspiration and desire for language learning (i.e., expectations).
*Ought-to L2 self* (six items; *α*_time1_ = 0.902; *α*_time2_ = 0.927; *α*_time3_ = 0.933; *α*_time4_ = 0.947; *α*_time5_ = 0.956; and *α*_time6_ = 0.942), concerns the attributes that one believes one should possess (i.e., responsibilities or obligations) in order to avoid possible negative outcomes.
*L2 learning experience* (six items; *α*_time1_ = 0.807; *α*_time2_ = 0.905; *α*_time3_ = 0.917; *α*_time4_ = 0.922; *α*_time5_ = 0.936; and *α*_time6_ = 0.932), measures learners’ attitudes, as well as situation-specific motives, related to the immediate learning environment and experience.
*Enjoyment* (10 items; *α*_time1_ = 0.904; *α*_time2_ = 0.935; *α*_time3_ = 0.911; *α*_time4_ = 0.931; *α*_time5_ = 0.928; and *α*_time6_ = 0.935), reflects two dimensions of foreign language enjoyment—private and social enjoyment—in a teacher-controlled classroom environment (Dewaele and MacIntyre, 2016).
*Anxiety* (eight items; *α*_time1_ = 0.801; *α*_time2_ = 0.847; *α*_time3_ = 0.882; *α*_time4_ = 0.898; *α*_time5_ = 0.885; and *α*_time6_ = 0.875), assesses the “degree of anxiety [in English], as evidenced by negative performance expectations and social comparisons, psychophysiological symptoms, and avoidance behaviors” (Horwitz and White, 1991, p. 37). Two items were phrased to indicate low anxiety and six items were phrased to reflect high anxiety (see Appendix A). The two low anxiety items were reverse-coded so that high scores on this measure reflect high anxiety.

According to the aforementioned reliability statistics, all the variables demonstrated high reliability and validity at each of the six waves.

### Procedures

The present study adopted a longitudinal design to identify salient patterns of Chinese learners’ motivational and emotional involvement in English learning. Specifically, an online questionnaire platform called Huixin was used to collect the data. Considering that motivation and emotions may vary in strength at different timescales (Dewaele and Dewaele, 2020), unique patterns of motivation and emotions might emerge at the macro level, which provide valuable and useful insights into motivation research. Unlike previous research (e.g., Waninge, 2015) that focused on changes at the micro level (i.e., lesson or week), the aim of present study was to create a macro-map (i.e., month) of motivation development. Specifically, we tend to show the temporal processes that occur over the course of months (Hollenstein, 2013). Thus, learners completed the same questionnaire at 2-month intervals over the course of 1 year (from October 2020 to August 2021), six times in total. The questionnaires were distributed purposefully to learners’ phones at English classes so that the first author would remind learners to complete questionnaires on time. The total number of the participants who completed the questionnaire was 198 at Time 1, 186 at Time 2, 183 at Time 3, 178 at Time 4, 180 at Time 5, and 176 at Time 6, respectively. Only data from participants who completed all six times were included in the present study; therefore, the total number of our participants was 176.

### Data Analysis

The data analysis consisted of four steps. In Step 1, we dealt with the first research question. To do so, a latent growth curve modeling (LGCM) technique was used, which identified the shape of growth of learners’ motivation and emotion over two semesters. Specifically, we followed the procedure in Jung and Wickrama (2008), and conducted and compared five unconditional models (i.e., without covariate) sequentially: intercept only, linear, quadratic, piecewise quadratic, and latent basis (freely estimated time) models to estimate the overall trajectory. Based on selection criteria such as comparative fit index (i.e., CFI > 0.95), Tucker-Lewis coefficient (i.e., TLI > 0.95), root mean square error of approximation (i.e., RMSEA < 0.06), and standardized root mean square residual (i.e., SRMR < 0.06; Mueller and Hancock, 2019), the model best fitted to the data was chosen.

Based on the results of LGCM, GMM was then estimated (Step 2), identifying the heterogeneity within a population and generating the number of classes for motivation and emotion, as well as the posterior probabilities of the class membership of each individual. We started with a single class model and then added classes to the model iteratively. To determine the optimal number of classes, Akaike Information Criterion (AIC), Bayesian information criterion (BIC), and sample-size-adjusted BIC (SSBIC), Entropy, the Adjusted Lo–Mendell–Rubin LRT (ALMRT), and the bootstrapped likelihood ratio test (BLRT) were all taken in account (Nylund et al., 2007; Wang and Bodner, 2007). Lower values of AIC, BIC, and SSBIC represent a better model fit. SSBIC is the most accurate criterion fit statistic for small samples (e.g., *N* < 500; Hensen et al., 2007). Higher values (0.70 or higher) of entropy indicate a more accurate classification of individuals into classes. In addition, significant value of *p* of ALMRT and BLRT indicate that the model with larger number of classes is favored (Nylund et al., 2007). Average posterior probabilities are also considered to assess the quality of the model solution, which indicate the individual’s probabilities of being assigned to each class based on their developmental pattern. Last, relevant theory and prior research were taken into consideration when selecting fitted models (Muthén, 2004). Thus, one-through four-class solutions were tested as estimating more than four classes would lead to convergence issues, concerning “principle of parsimony” of model selection and relatively small sample size in present study.

Since covariates would influence the growth parameters (intercept or slope), a conditional GMM can be estimated with greater accuracy, including the covariates could specify additional information on class membership for motivation and emotions (Lubke and Muthén, 2007). Previous studies have shown that gender is correlated with learning motivation and emotion (Dewaele and MacIntyre, 2014; Dewaele et al., 2016). For example, Dewaele et al. (2016) examined the role gender played in influencing FL learners’ anxiety and enjoyment with the same dataset. It was found that female learners enjoyed the FL class more than male learners. However, different from the results in previous research (e.g., Dewaele et al., 2016), Dewaele and Dewaele (2017) found that gender had a limited effect on emotions. Due to the confounding results regarding the role of gender in emotions, further exploration is warranted. Thus, we recognized gender as a covariate of the GMM model (Step 3) and examined the association between gender and the class membership with a multinomial logit model. Odds ratio (OR) was adopted to explain whether the probability of obtaining a high level of services is the same for two classes. Specifically, An OR of 1 means that exposure to the first class does not affect the odds of the second. An OR of more than 1 means that a greater likelihood for the first class to receive high services. An OR is less than 1 is associated with lower odds.

Step 4 concerned the second and the third research questions, for which we conducted a parallel-process GMM to explore the temporal relationship between motivation and emotions (anxiety and enjoyment). Specifically, we examined the joint probability and conditional probability of that individual being in each motivation trajectory subgroup membership in a specific emotion trajectory, and the probability of being in each emotion trajectory subgroup membership in a specific motivation trajectory. That is, this novel method models motivation and emotion latent growth classes simultaneously as two separate growth processes instead of a single process with multiple indicators. All these models were performed on Mplus 8.6.

## Findings

### Salient Patterns of Motivation

The results of unconditional LGCM analysis (see Table 1) showed that the intercept-only, linear, quadratic, and piecewise quadratic models provided poor fits to the data, and that only the latent basis (freely estimated time) model of growth had good model fit statistics (CFI = 1, TLI = 1, RMSEA = 0.00, and SRMR = 0.04). Thus, it is clear that the latent basis model would serve as the best base model for growth mixture analyses. As one of the growth models, the latent basis model can describe nonlinear changing patterns and is flexible to determine the shape of change best fitting the participants’ data across time (Duncan et al., 2006; Ram and Grimm, 2007). Based on the unconditional LGCM, the mean trajectory differed significantly from zero and is positive (Intercept = 3.75, *p* < 0.001; Slope = 0.03, *p* < 0.001), indicating that the mean trajectory developed in a non-linear manner with an increasing growth pattern.

**Table 1:** Fit statistics for LCGM models.

Model of Change	CFI	TLI	RMSEA	SRMR	*χ* ^2^
**Motivation**					
Intercept	0.51	0.61	0.21	0.52	160.57
Linear	0.74	0.76	0.16	0.24	90.06
Quadratic	0.99	0.99	0.04	0.07	14.80
Piecewise	0.97	0.97	0.06	0.10	19.90
**Latent basis**	**1.00**	**1.00**	**0.00**	**0.04**	**11.81**
**Enjoyment**					
Intercept	0.64	0.71	0.16	0.38	106.14
Linear	0.88	0.89	0.10	0.12	44.66
Quadratic	0.99	0.98	0.04	0.06	15.48
Piecewise	0.98	0.97	0.05	0.07	17.38
**Latent basis**	**0.99**	**0.98**	**0.04**	**0.04**	**16.21**
**Anxiety**					
Intercept	0.58	0.67	0.17	0.36	116.80
Linear	0.86	0.87	0.11	0.15	48.80
Quadratic	0.99	0.98	0.04	0.08	15.54
Piecewise	0.92	0.90	0.10	0.13	31.21
**Latent basis**	**1.00**	**1.00**	**0.00**	**0.03**	**10.10**

fit index > 0.95 (CFI), Tucker-Lewis coefficient > 0.95 (TLI), root mean square error of approximation < 0.06 with a confidence interval upper limit of <0.08 (RMSEA with CI 90%), and standardized root mean square residual of <0.06 (SRMR). The bold values highlights that latent basis model of growth had good model fit statistics.

After determining the optimal number of classes, the GMM models for 1-, 2-, 3-, and 4-classes were assessed successively (see Table 2). We had attempted to fit a four-class model, for the likelihood ratio tests were still significant and variance within and cross classes remained. However, several warning messages indicated that the four-class model was not feasible. Considering that there were only six participants assigned to class 4 and that the proportion in each of the latent classes should be at least 5%, we did not improve convergence. Given the lower AIC, BIC, and SSA-BIC values, significant likelihood ratio tests, and the third-class solution had the best fit as compared to 1-class and 2-class solutions. Therefore, the 3-class solution was retained.

**Table 2:** Fit information for the GMM.

No. of classes	Log likelihood	AIC	BIC	SSBIC	Entropy	A-LRT	BLRT	*N*
						*p*-value	*p*-value	
*Motivation*								
1 Class	−1072.46	2170.93	2212.14	2170.98	N/A	N/A	N/A	176
2 Class	−1014.17	2062.35	2116.25	2062.41	0.73	0.02	<0.001	123;53
3 Class	**−977.78**	**1995.59**	**2059**	**1995.67**	**0.84**	**0.05**	**<0.001**	11;48;117
*Enjoyment*								
1 Class	−1063.24	2152.48	2193.7	2152.53	N/A	N/A	N/A	176
2 Class	**−1010.43**	**2054.85**	**2108.75**	**2054.92**	**0.87**	**0.01**	**<0.001**	**151;25**
3 Class	−991.26	2024.52	2091.1	2024.6	0.86	0.23	<0.001	22;56;98
*Anxiety*								
1 Class	−1115.44	2256.87	2298.09	2256.92	N/A	N/A	N/A	176
2 Class	**−1073.74**	**2181.47**	**2235.37**	**2181.54**	**0.83**	**0**	**<0.001**	**31;145**
3 Class	−1054.73	2151.47	2218.05	2151.55	0.72	0.65	<0.05	37;79;60

*N* = 176. AIC, Akaike information criterion; BIC, Bayesian information criterion; SSABIC, sample-size adjusted Bayesian information criterion; Adjusted LRT, Lo–Mendell–Rubin Adjusted Likelihood Ratio Test; and BLRT, bootstrapped likelihood ration test. The values shown in bold indicate the model with the smallest value. *N* = estimated number of individuals in each class.

**Figure 1:**
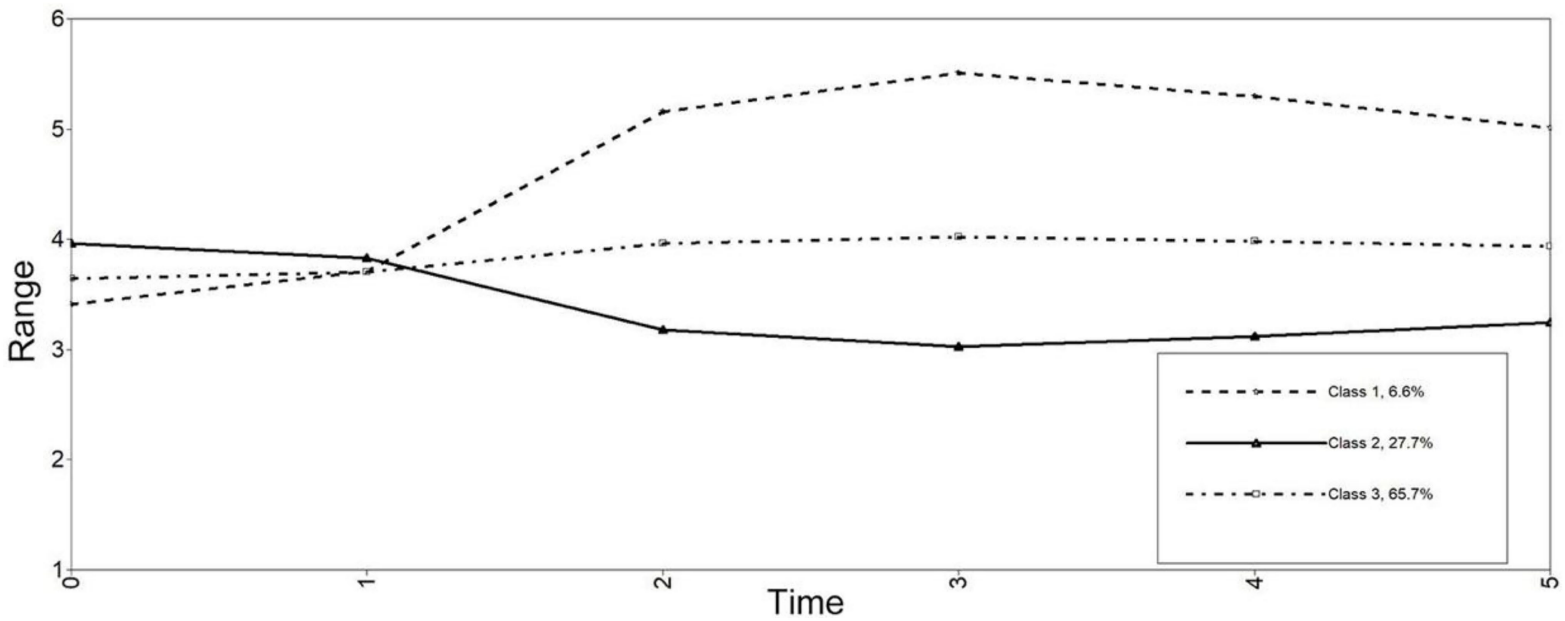
Estimated longitudinal trajectories of motivation for 3-class.

Class 1 termed as *the moderate increase class* (6.6%) initially had moderate levels of motivation (intercept = 3.41, *p* < 0.001) and showed gradual increase to a higher level (slope = 1.60, *p* < 0.001). Class 2, *the slight decrease class* (27.6%) who initially had high levels of motivation (intercept = 3.96, *p* < 0.001), but decreased slightly with fluctuation over time (slope = −0.72, *p* < 0.001). Class 3, *the highly stable class* (65.7%) who had high-stable level of motivation over time (intercept = 3.65, *p* < 0.001; slope = 0.29, *p* < 0.001; See Figure 1). OR revealed that gender was not a significant predictor for class membership (OR = 0.49, 95%CI: 0.24–1.02).

### Salient Patterns of Enjoyment

For the overall trajectory of enjoyment, the unconditional latent basis LGCM also yielded better model fit statistics (CFI = 0.99, TLI = 0.98, RMSEA = 0.04, and SRMR = 0.04) than others (see Table 1). Based on the unconditional LGCM, the estimated mean value of enjoyment was 4.37 (*p* < 0.001), and decreased slightly over time (Slope = −0.09, *p* < 0.001). Based on the unconditional latent basis LGCM of enjoyment, GMM was adopted to identify the heterogeneity (see Table 2). Given the lower AIC, BIC, and SSA-BIC values, significant likelihood ratio tests, the two-class solution had the best fit as compared to 1-class and 3-class solutions. Therefore, the 2-class solution was chosen here.

**Figure 2:**
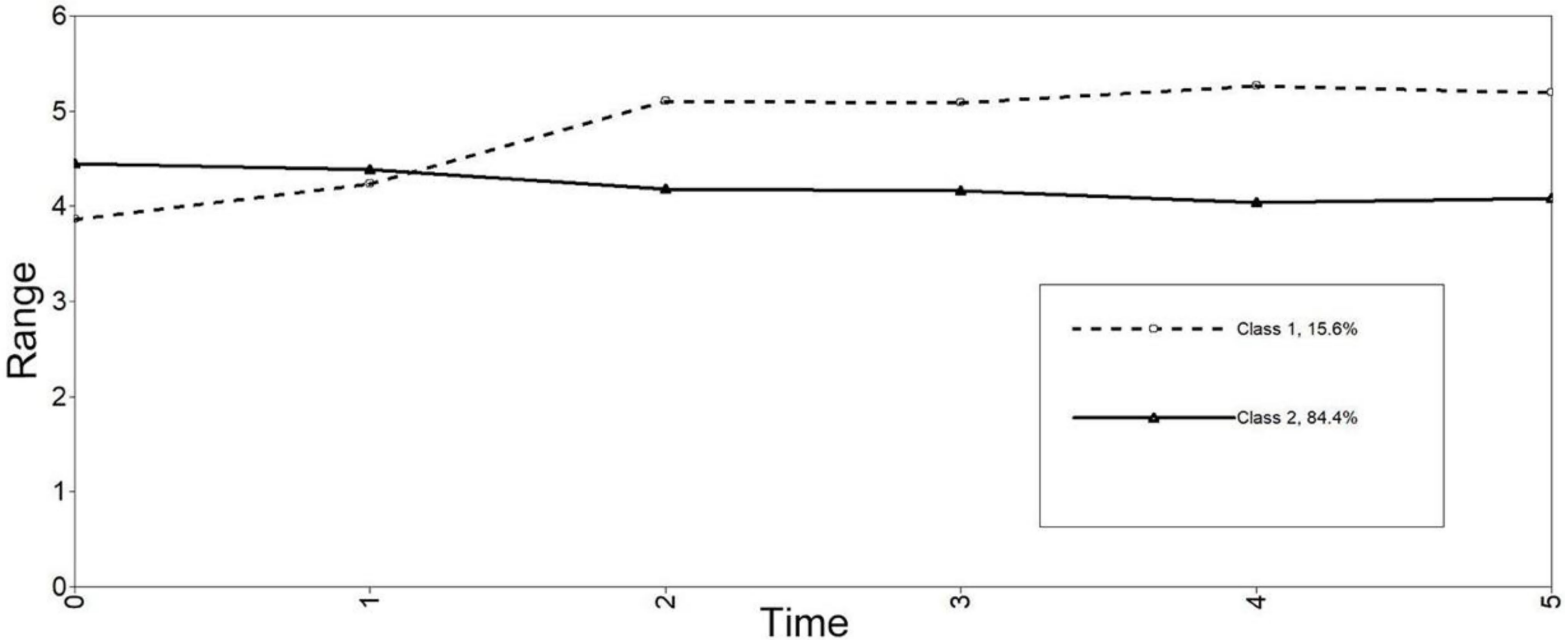
Estimated longitudinal trajectories of enjoyment for 2-class.

Class 1, *the gradual increase class* (15.6%) had a high-stable level of enjoyment over time. Specifically, Class 1 had a moderate starting point (intercept = 3.89, *p* < 0.001) and positive growth (slope = 1.33, *p* < 0.001). Class 2, *the slight increase class* (84.4%), who initially had high levels of enjoyment (intercept = 4.47, *p* < 0.001), and decreased slightly at the later stage (slope = −0.37, *p* < 0.001; see Figure 2). OR revealed that gender was not a significant predictor for class membership (OR = 1.613, 95%CI: 0.57–4.55).

### Salient Patterns of Anxiety

For anxiety, the unconditional latent basis LGCM also yielded better model fit statistics (CFI = 1, TLI = 1, RMSEA = 0.00, and SRMR = 0.03) than others (see Table 1). Based on the unconditional LGCM, the estimated mean value of anxiety was 3.98 (*p* < 0.001), and decreased over time (Slope = −0.90, *p* < 0.001). Based on the unconditional latent basis LGCM of anxiety, GMM was adopted to identify the heterogeneity (see Table 2). Compared to 1-class and 2-class solutions, the 3-class solution had the best fit with lower AIC, BIC, and SSA-BIC values, significant ALMRT and BLRT. Therefore, the 3-class solution was selected.

**Figure 3:**
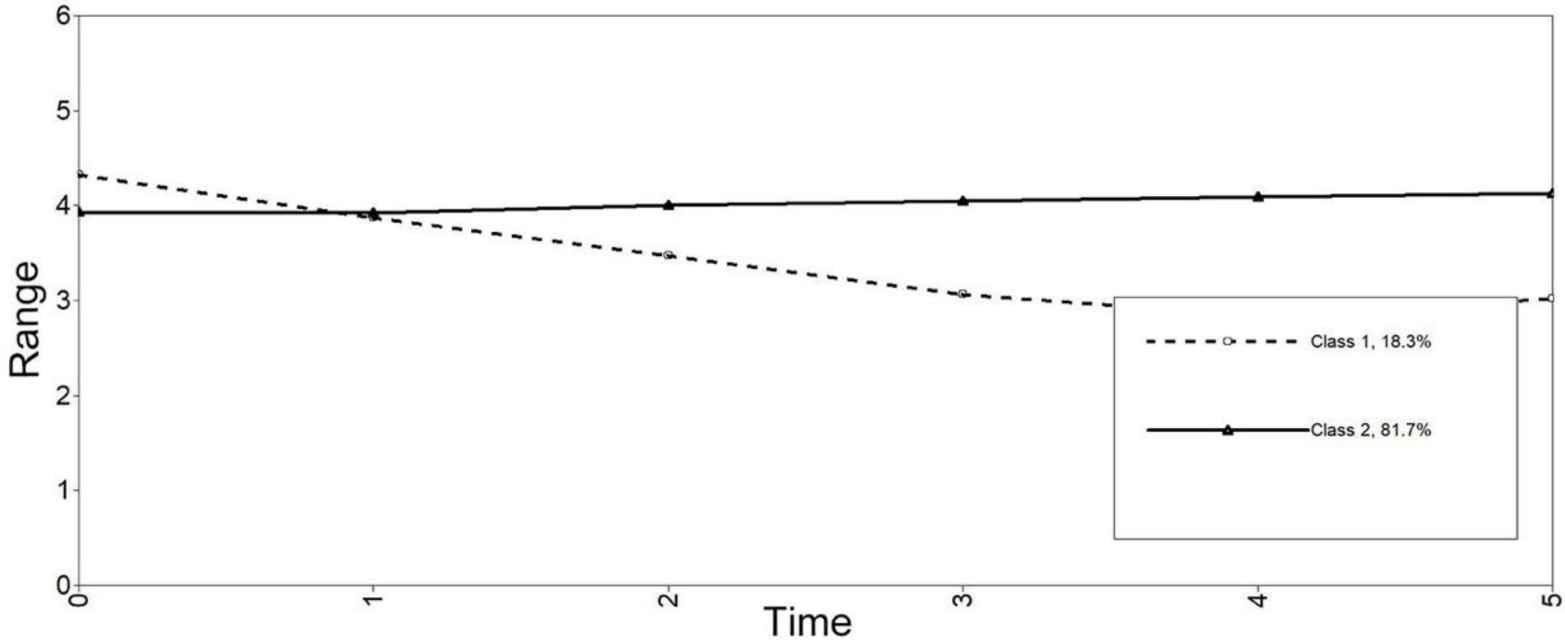
Estimated longitudinal trajectories of anxiety for 2-class.

Class 1, *the moderate decrease class* (89.3%), had a moderate starting point (intercept = 4.30, *p* < 0.001) and decreased moderately at the later stage (slope = −1.31, *p* < 0.001). Class 2, *the high stable class* (10.7%), had high stable levels of anxiety (intercept = 3.90, *p* < 0.001), and slightly increased over time (slope = 0.19, *p* < 0.001; see Figure 3). OR revealed that gender was not a significant predictor for class membership (OR = 1.30, 95%CI: 0.58–2.91).

### Joint Group Membership Between Subgroups of Motivation and Emotions

By adopting a parallel process GMM technique, the joint probabilities of class membership for the growth mixture classes from the two processes were obtained for all participants. Table 3 shows the joint and conditional probabilities for motivation and emotions (enjoyment and anxiety).

**Table 3:** Joint and conditional probabilities for motivation and emotions.

Enjoyment	Motivation		
	Moderate increase	Slight decrease	High stable
*Joint Probability (frequency)*			
Gradual increase	0.14 (25)	0.69 (122)	0
Slight decrease	0.06 (11)	0.10 (18)	0
			
*Conditional probability*			
Gradual increase	0.71	0.85	0
Slight decrease	0.29	0.15	0.
**Anxiety**	**Motivation**		
	**Moderate increase**	**Slight decrease**	**High stable**
*Joint Probability (frequency)*			
Moderate decrease	0.07 (12)	0.02 (4)	0
High stable	0.06 (10)	0.08 (14)	0.77 (136)
			
*Conditional probability*			
Moderate decrease	0.48	0.28	0
High stable	0.52	0.72	1

For enjoyment, out of 176 learners, only 43 (24.6%) had joint (simultaneous) membership in the similar type of enjoyment and motivation subclasses. Specifically, 25 (14.2%) in the (*gradual or moderate*) *increase class* and 18 (10.2%) in the *slight decrease class*. It should be noted that no learners were simultaneously in the *high stable* class for motivation and the *gradual increase*, *slight decrease* class for enjoyment. According to the conditional probabilities of simultaneously being in an enjoyment class and in a motivation latent class shown in Table 3, among the individuals in the *gradual increase class* for enjoyment, the likelihood of also being in the *moderate increase* motivation class was 0.71, compared to the likelihood of being in the *slight decrease* motivation class (0.85) and of being in the *high stable* motivation class (0.49). Learners in the *slight decrease* enjoyment class had a 0.29 probability of also being in the *moderate increase* motivation class, only a 0.15 likelihood of being in the *slight decrease* motivation class, and a 0.51 likelihood of being in the *high stable* motivation class. Further, based on the estimated probabilities, the OR showed that when compared to those in the *slight decrease* enjoyment, those in the *gradual increase* enjoyment were 0.33 times more likely to be in the *slight decrease* motivation relative to being in the *moderate increase* motivation (OR = 0.33, 95%CI 0.14–0.80, *p* < 0.05).

For anxiety, out of 176 learners, 141 (80%) had joint (simultaneous) membership in the similar type of anxiety and motivation subgroups, and they all came from the same type of *high stable*. It should be noted that no learners were simultaneously in the *high stable* for motivation and *moderate decrease* for anxiety. With regard to the conditional probabilities of membership in an anxiety class given membership and in a motivation latent class, learners in the *moderate decrease* anxiety class had a 0.48 probability of also being in the *moderate increase* motivation class, while none of the learners in the *slight decrease* motivation class, nor in the *high stable* motivation classes. The learners in the *high stable* anxiety class were all in the *high stable* motivation class at the same time, while having only a 0.52 probability of being in the *moderate increase* motivation class. Moreover, the OR showed that when compared to learners in the *moderate decrease* anxiety, those in the *high stable* anxiety were 4.20 times more likely to be in the *slight decrease* motivation relative to being in the *moderate increase* motivation (OR = 4.20, 95%CI 1.04–16.90, *p* < 0.05).

## Discussion

The current study, with the application of a novel parallel-process GMM approach, serves as an initial attempt to identify typical trajectories of foreign language learners’ learning motivation and emotion (i.e., enjoyment and anxiety) development, and to distil salient patterns of the motivation–emotion association over time.

### Salient Patterns

With regard to RQ1, we identified three salient classes with distinct developmental patterns for language learning motivation and two developmental patterns for emotion, respectively, among the 176 Chinese college students over two semesters. This observation highlighted a particular contribution of the GMM technique to motivation research; that is, it helped to identify patterned outcomes regarding the complex and dynamic processes of motivational development, which might have been ignored in most process-oriented research where *inter-individual differences in terms of developmental processes* were not considered (e.g., at a relatively global, subgroup level; see similar views in Baba and Nitta, 2021; Lee and Ju, 2021). Specifically, three different classes (each with a distinct developmental pattern) were found for the 176 Chinese EFL learners’ learning motivation, with the *high stable* class comprising the dominant developmental pattern (65.7%), corroborating the finding of Guay et al. (2021) that most learners had constantly high levels of motivation over time. However, different from the three motivation patterns identified here, Guay et al. (2021) observed five different profiles of motivation development. This discrepancy may be partly ascribed to the different learners focused on. The present study targeted college-level students who are more likely to have limited or stable motivation, as noted in Guay et al. (2021), whereas secondary school students with diversified motivational characteristics were examined in Guay et al. (2021).

It is important to note that three typical profiles with distinct motivational trajectories (i.e., *increase*, *high stable*, *and decrease*) were both found in Guay et al. (2021) and in the present study. This identification of typical motivation profiles adds empirical credence to the view of Papi and Teimouri (2014) that similar motivational patterns would emerge from data and can hold for learners from different socio-educational contexts. Based on this finding, we may conclude that learners’ learning motivation tend to follow three general trajectories over time, and tailored teaching strategies should thus be designed and implemented to scaffold learners for their learning motivation enhancement.

With regard to learners’ emotions, two distinct classes were identified for enjoyment and for anxiety, respectively. For enjoyment, most learners (84.4%) showed increased enjoyment over time, indicating that the majority of learners were increasingly enjoying English learning in university and showed a positive attitude toward English learning. For anxiety, most learners (89.3%) showed decreased anxiety over time. In other words, a large number of students changed their negative attitudes toward English learning over time. The changing processes of enjoyment and anxiety observed in the present study corroborated the view of Dewaele and Dewaele (2017) that learners’ emotions (enjoyment and anxiety) are malleable and dynamic in nature. Moreover, the identification of different developmental patterns (i.e., classes) for enjoyment and for anxiety, respectively, rendered further support to the recent proposal that enjoyment and anxiety are best regarded as two distinct, independent emotions not following a single and simple pattern (Dewaele and MacIntyre, 2014; Boudreau et al., 2018), and should therefore be conceptualized and empirically examined separately.

By using a parallel-process GMM technique, the present study managed to examine the developmental processes of learning motivation and emotions (which are often idiosyncratic at the individual level) at a relatively higher and global level (i.e., the subgroup level). That is, the present study identified salient patterns of motivational and emotional development over time, providing important insights into the inter-individual differences in intraindividual changes, and increased, to some degree, the generalizability of CDST-inspired, process-oriented research findings which often reveal idiosyncratic, dynamic features of individual development (Baba and Nitta, 2021; Peng et al., 2022).

It should be noted that, in our application of the GMM technique, we also included gender as a covariate, results of which showed that gender did not affect the change and development of EFL learners’ motivation and emotions (both enjoyment and anxiety) significantly. In other words, the present study, by employing a longitudinal design (i.e., lasting 1 year) and adopting a quantitative analytic approach (i.e., using GMM technique), provided empirical evidence to the finding of Dewaele and Dewaele (2017) that gender would only exert a limited effect on the development of language learners’ learning motivation and emotions. In light of this observation, we would recommend future research on language learners’ learning motivation and emotion pay more attention to the role of other learner-related (e.g., language proficiency) or task-related factors (e.g., task complexity) in influencing the development of language learning motivation and emotions.

### The Association Between Motivation and Emotions

A more important contribution of the present study is that it provided empirical evidence for the intimate connection and adaptive interaction between language learning motivation and emotions (enjoyment, anxiety; RQ2). Namely, membership in motivation trajectory classes was found in close association with membership in certain emotions trajectory classes. More specifically, enjoyment and anxiety were found both significantly affecting the development of learning motivation, but in different ways. In terms of the anxiety-motivation relation, we found a strong, positive connection between the two, partly echoing Papi and Teimouri’s (2014) view that anxiety plays a facilitative role in learners’ prevention-focused motivation (e.g., ought-to L2 selves). It is worth noting that the participants (i.e., Chinese EFL learners) targeted in the present study were more likely to be driven by their ought-to L2 selves closely related to the prevention-focused motives, as English language education in China is often exam-oriented (Peterson et al., 2013). A similar view was also observed in Teimouri (2017), which found that “L2 anxiety fits the motivational orientation of learners with a predominant preventional focus and plays a facilitative role by keeping them alert to the presence of possible negative outcomes” (p. 702).

With regard to the enjoyment-motivation relation, we observed a negative association between them. This was an unexpected observation, as previous studies have reported a positive relationship between learning motivation and enjoyment (e.g., Teimouri, 2017; Pan and Zhang, 2021; Papi and Khajavy, 2021). The discrepancy between the present study and prior research might be related to the temporal dimension focused on and the specific learning context (Chinese EFL learning context) in which the study was situated in. For example, the data reported in the present study were collected over a time period of two semesters, during which online and offline courses were adopted alternately or in combination. This changing teaching strategy may have led to our participants’—Chinese EFL learners—less motivated involvement in English learning. At the same time, this teaching strategy used may also have bolstered their enjoyment of English learning, as, by taking online courses, they no longer needed to communicate with teachers and peers directly and hence avoided the possible embarrassment of speaking in class, as García-Alberti et al. (2021) noted. A pedagogical implication from this is that educators may consider designing and conducting diverse learning tasks and activities to engage, to motivate, and to support Chinese EFL learners as so to boost their English learning motivation and enjoyment. Further research is needed to systematically explore the underlying reasons that cause this negative motivation–enjoyment association, preferably with a quantitative–qualitative mixed design.

This unexpected relation between motivation and enjoyment might also be related to the specific participants targeted in the present study (i.e., college-level non-English majors). Despite showing positive attitudes toward English learning, non-English majors’ motivation was often not strong, because they have their own specialties and learning tasks, and also because college English tests (CET 4 or 6) were no longer tied to their bachelor degree. The negative relationship between enjoyment and motivation testified that learners who enjoyed English learning may have a weaker motivation to learn it. A possible pedagogical implication of this observation is that learning context as an important component of the complex language learning system should be constantly revisited when educators consider supporting and enhancing language learners’ learning motivation and emotions. A related implication is that, given the patterned interactions observed between learning motivation and emotions, educators should also find ways to scaffold language learners’ learning motivation through designing, implementing, and assessing specific learning activities so as to boost learners’ enjoyment while relieving their anxiety.

Combined, the observed patterns of the adaptive interaction between learning motivation and emotions (enjoyment and anxiety) lent further support to the increasing recognition that motivation is a relational system, in which subsystems interconnect in ways that lead to new regimes of order over time (Hiver et al., 2021; Larsen-Freeman, 2021). And the unique and unexpected interactions between motivation and enjoyment (i.e., a negative relation) further corroborated that language learning motivation is often situated and contextually constrained, which in turn help to shape motivation’s interaction with learners’ emotion in learning (another) language (Larsen-Freeman, 2021).

## Conclusion

The present study applied an innovative parallel-process GMM method to motivation and emotion research, which generated novel results concerning motivation–emotion coordination over time. Specifically, the study revealed salient patterns of motivational and emotional development, as well as the adaptive interactions between learning motivation and emotions over a period of 12 months. First, three profiles of motivation development and two profiles of emotion development were identified, respectively. Additionally, gender could not predict the development of both motivation and emotions. Second, both enjoyment and anxiety showed significant associations with learning motivation. A positive relation was also found between anxiety and motivation. However, different from previous findings, a negative relation between enjoyment and motivation was observed.

These findings enriched our understanding of the dynamic evolvement of learning motivation and emotions over time at the group level, and provided a holistic, detailed account of the motivation–emotion associations. What is more, the parallel-process GMM has been proved to be an applicable and significant approach to parsing heterogeneity within a sample so as to identify the salient patterns that meaningfully summarized the complex, constantly changing processes of language learning motivation and emotions, results of which are of practical and pedagogical significance. Given that the present study analyzed the data only with quantitative methods, future studies may consider exploring and validating typical profiles of motivational and emotional development using a quantitative–qualitative mixed approach. Moreover, a set of covariates (i.e., learning achievement and age) should be taken into account to predict the development of language learning motivation and emotions, which may provide a systematic and valuable explanation for the change and development of learning motivation and emotions, and help educators adopt practical strategies to intervene the complex dynamic systems actively.

## Data Availability Statement

The raw data supporting the conclusions of this article will be made available by the authors, without undue reservation.

## Author Contributions

HY: conceptualization, data curation, formal analysis, funding acquisition, investigation, methodology, project administration, and writing—original draft. HP: conceptualization, data curation, methodology, project administration, and writing—review and editing. WL: conceptualization, supervision, and writing—review and editing. All authors contributed to the article and approved the submitted version.

## Funding

This research was supported by the Fundamental Research Funds for the Central Universities, Dalian University of Technology (DUT22RW126).

## Conflict of Interest

The authors declare that the research was conducted in the absence of any commercial or financial relationships that could be construed as a potential conflict of interest.

## Publisher’s Note

All claims expressed in this article are solely those of the authors and do not necessarily represent those of their affiliated organizations, or those of the publisher, the editors and the reviewers. Any product that may be evaluated in this article, or claim that may be made by its manufacturer, is not guaranteed or endorsed by the publisher.
